# Neuroplasticity related to chronic pain and its modulation by microglia

**DOI:** 10.1186/s41232-022-00199-6

**Published:** 2022-05-03

**Authors:** Shin-ichiro Hiraga, Takahide Itokazu, Mariko Nishibe, Toshihide Yamashita

**Affiliations:** 1 Department of Neuro-Medical Science, Graduate School of Medicine, Osaka University, Suita, Osaka Japan; 2 Department of Molecular Neuroscience, Graduate School of Medicine, Osaka University, Suita, Osaka Japan; 3 Center for Strategic Innovative Dentistry, Graduate School of Dentistry, Osaka University, Suita, Osaka Japan; 4 WPI Immunology Frontier Research Center, Osaka, Japan; 5 Graduate School of Frontier Biosciences, Osaka University, Osaka, Japan

**Keywords:** Nociplastic pain, Neuroplasticity, Microglia, PNS injury, CNS injury, Thalamic hemorrhage

## Abstract

Neuropathic pain is often chronic and can persist after overt tissue damage heals, suggesting that its underlying mechanism involves the alteration of neuronal function. Such an alteration can be a direct consequence of nerve damage or a result of neuroplasticity secondary to the damage to tissues or to neurons. Recent studies have shown that neuroplasticity is linked to causing neuropathic pain in response to nerve damage, which may occur adjacent to or remotely from the site of injury. Furthermore, studies have revealed that neuroplasticity relevant to chronic pain is modulated by microglia, resident immune cells of the central nervous system (CNS). Microglia may directly contribute to synaptic remodeling and altering pain circuits, or indirectly contribute to neuroplasticity through property changes, including the secretion of growth factors. We herein highlight the mechanisms underlying neuroplasticity that occur in the somatosensory circuit of the spinal dorsal horn, thalamus, and cortex associated with chronic pain following injury to the peripheral nervous system (PNS) or CNS. We also discuss the dynamic functions of microglia in shaping neuroplasticity related to chronic pain. We suggest further understanding of post-injury ectopic plasticity in the somatosensory circuits may shed light on the differential mechanisms underlying nociceptive, neuropathic, and nociplastic-type pain. While one of the prominent roles played by microglia appears to be the modulation of post-injury neuroplasticity. Therefore, future molecular- or genetics-based studies that address microglia-mediated post-injury neuroplasticity may contribute to the development of novel therapies for chronic pain.

## Introduction

Injuries to neural circuits, whether in the peripheral or central somatosensory system, can result in neuropathic pain, an intractable chronic pain manifests as an array of symptoms and signs [1]. The prevalence of chronic pain increases with age, and pain is associated with decreased quality of life, opioid dependence, and poor mental health. The International Association for the Study of Pain has recently proposed a new mechanism of pain development termed nociplastic pain, a type of pain that occurs as a result of plastic changes in the neural circuits carrying nociceptive information [2]. Nociplastic pain is a third type of pain, apart from the two pain mechanisms, which are nociceptive pain (caused by tissue damage or threaten tissue damage) and neuropathic pain (caused by damage to the peripheral nervous system [PNS] or central nervous system [CNS]). Although nociplastic pain can manifest solely, the symptoms often occur as part of a mixed state with the other two mechanisms [2]. To regulate pain occurrence, it is important to understand whether the pathophysiology of pain is a direct result of nervous system trauma or a result of neuroplastic changes secondary to trauma to tissues or to neurons. Recently, microglia, the resident immune cells of the CNS, have emerged as a key modulator of neurons in regulating synaptic pruning, formation, and transmission, thereby contributing to the pathophysiology of pain [3]. The role of microglia in the spinal dorsal horn in modulating neuropathic pain has been well studied compared to the role of intracerebral microglia [4]. We reported the involvement of intracerebral microglia in the development of chronic pain associated with the alteration of nociceptive neural circuitry post-CNS injury [5]. In this review, we highlight the neuroplastic mechanisms that occur in somatosensory circuits in the spinal dorsal horn, thalamus, or cortex associated with chronic pain, following injury to the PNS or CNS. We then discuss the dynamic functions of microglia in shaping neuroplasticity related to chronic pain.

## Neuroplasticity of spinal dorsal horn interneurons in chronic pain following a PNS injury

The dorsal horn of the spinal cord, organized into six Rexed laminae and composed broadly of excitatory and inhibitory neurons, gathers sensory information of the peripheral tissues through the afferent sensory neurons. Nociceptive information is transmitted to the superficial laminae I and II of the dorsal horn, and non-nociceptive information is transmitted to the deep laminae II–VI. Integrated sensory information is then relayed to several brain regions, including the thalamus. When a peripheral injury occurs to an extent that alters the somatosensory information processing system, it often leads to evoking pain sensation by normally non-noxious stimuli. Such an alteration in pain perception is often induced by structural and functional neuroplasticity of the spinal dorsal horn due to its closely related anatomical structures of each lamina. Pain evoked by non-noxious stimuli is called allodynia and it is one of the characteristics of neuropathic and nociplastic pain.

**Fig. 1:**
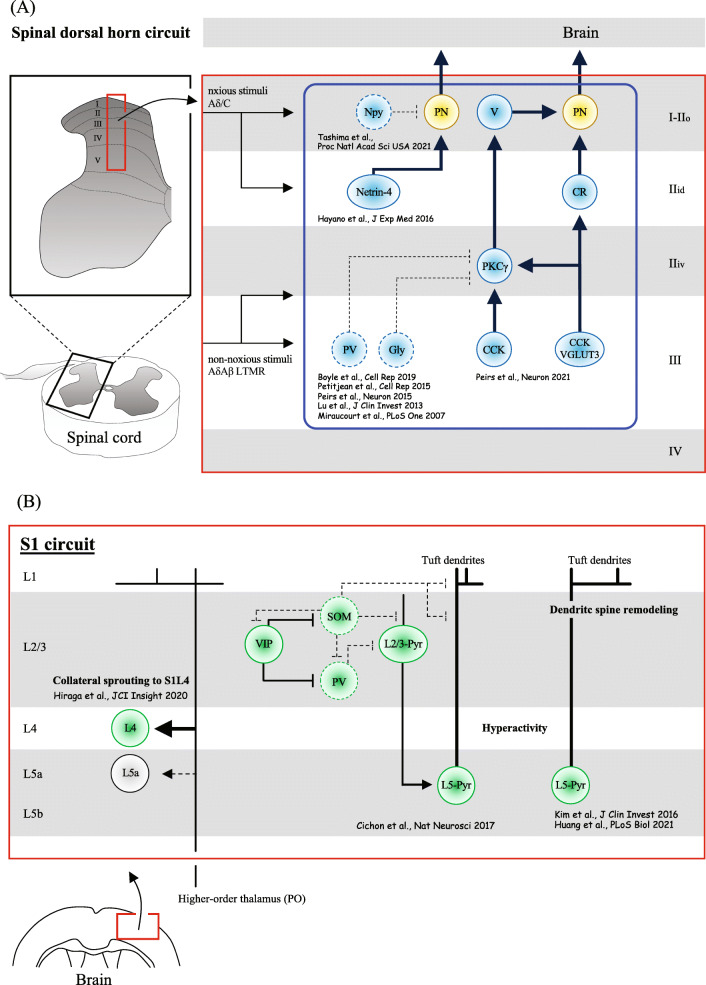
The spinal dorsal horn and S1 neuroplasticity mechanism inducing chronic pain following PNS or CNS injury. **A** Synaptic modifications in the dorsal horn of the spinal cord result in a balance alteration of excitatory and inhibitory synaptic transmission in lamina I projection neurons to the brain, which account at least partially for pain sensation evoked by non-noxious stimuli. The continuous lines and arrows indicate allodynia-relevant circuits while dotted lines indicate neuronal activity suppression or synaptic loss in the cause of enhanced pathology. Arrowheads indicate the direction of excitatory inputs. PV, parvalbumin-expressing neurons; SOM, somatostatin-expressing neurons; CCK, cholecystokinin-expressing neurons; VGLUT3, vesicular glutamate transporter 3-expressing neurons; PKCγ, protein kinase C gamma-expressing neurons; CR, calretinin-expressing neurons; Gly, glycine-expressing neurons; Netrin-4, netrin-4-expressing neurons; Npy, neuropeptide Y inhibitory interneurons; PN, projection neuron; LTMR, low-threshold mechanoreceptors; V, vertical cells; IIo, outer lamina II, IIi, inner lamina II; IIid, inner lamina II (dorsal); IIiv, inner lamina II (ventral); Aβ, Aβ myelinated fibers; Aδ, Aδ myelinated fibers; C, C fibers. **B** Axonal sprouting and circuit functional reorganization of S1 involving both intracortical neurons and projection neurons are at least partially responsible for inducing pain by non-noxious stimuli. The continuous lines and arrows indicate allodynia-relevant circuits, and dotted lines indicate suppressed neuronal activity or synaptic losses in the cause of enhanced pathology. Arrowheads indicate the direction of excitatory inputs. White circle (L5a) indicates decreased neuronal hyperactivity after injury. L, layer; Pyr, pyramidal neurons; PV, parvalbumin-expressing neurons; SOM, somatostatin-expressing interneurons; VIP, vasoactive intestinal polypeptide-expressing interneurons; PO, neurons from the thalamic posterior nucleus

The excitatory and inhibitory neurons in the spinal dorsal horn have been morphologically and electrophysiologically analyzed, and their subtypes have also been identified by molecular and gene expression profiles [6–11]. These findings promote the understanding of neuroplasticity occurring in interneurons of the spinal dorsal horn circuit, which is associated with allodynia after PNS injury. Novel pain circuits relevant to neuropathic and nociplastic pain are still being discovered within the dorsal horn (Fig. 1A). For instance, the gamma isoform of protein kinase C (PKCγ) interneurons, defined by the expression of Tac2, neurotensin, and cholecystokinin (CCK) [12] localized within the lamina II_iV_/III border and partially in laminae I and deeper III, have been shown to mediate mechanical allodynia induced by neuropathic injuries. These pain mediations were shown to be through PKCγ neurons losing at least a partial synaptic connection with parvalbumin or with glycine inhibitory neurons [12–17], and partially through interactions with 5-hydroxytryptamine receptor 2A (5-HT2A) [18]. This subtype of neurons differs from calretinin (CR) neurons located in the inner lamina II (IIi), which mediates mechanical allodynia induced by complete Freund’s adjuvant (CFA)-elicited inflammatory injury. Commonly affected by both neuropathic and inflammatory injuries, a subset of CCK neurons composed of transient vesicular glutamate transporter 3 (tVGLUT3) neuron, is involved in developing dynamic mechanical allodynia, making synaptic connections with PKCγ neurons. This VGLUT3/CCK/PKCγ circuit ultimately sends the input to the projection neurons in lamina I through vertical cells [16].

Another well-studied neuroplastic change associated with allodynia occurs in neuropeptide Y promoter (Npy) inhibitory interneurons [19]. The Npy inhibitory interneurons in the outer lamina II (II_o_) send inhibitory gamma-aminobutyric acid (GABA) signals to projection neurons in lamina I. The excitability of Npy neurons decreased after a PNS injury, inducing touch-evoked pain-like behavior, which improved by increasing the excitability of the interneurons. Notably, the axons of Npy neurons project to lamina III/IV cells, including PKCγ neurons [20], the subtype of interneurons that is closely associated with noxious stimuli. Our research group has identified that Netrin-4, a laminin-related extracellular protein, was expressed exclusively in the excitatory interneurons of the lamina IIi, mediating tactile and heat hyperalgesia following a PNS injury [8]. Neurophysiological experiments showed that Netrin-4 neurons induced pain sensation through an increase in the excitatory transmission of N-methyl-D-aspartic acid (NMDA) receptors. Together, synaptic modifications in the dorsal horn circuits of the spinal cord result in a balance alteration of synaptic transmission of excitation and inhibition in lamina I projection neurons to the brain, which account at least partially for the development and maintenance of chronic pain.

## Neuroplasticity of thalamocortical (somatosensory cortex) circuit in chronic pain after a PNS or CNS injury

Noxious and non-noxious stimuli applied to peripheral tissues are conveyed to the brain via the spinal cord mainly by the projection neurons in lamina I and V of the spinal dorsal horn [21]. The projection neurons in lamina I transmit nociceptive information to the periaqueductal gray matter (PAG), lateral parabrachial area (LPb), and ventral posterolateral nucleus (VPL) of the thalamus. The majority of the projection neurons in lamina V also terminate in the thalamic VPL nucleus and convey non-noxious stimuli. Therefore, the thalamic VPL nucleus, the “first-order nucleus,” serves as a gateway that processes both noxious and non-noxious stimuli to the fourth layer of the primary somatosensory cortex (S1L4). The inputs from the S1 are sent to the fourth layer of the secondary somatosensory cortex (S2L4) via the posterior nucleus (PO) of the thalamus, the nucleus is thus considered the “higher-order nucleus.” The PO nucleus is more heavily involved in noxious stimuli mediation projecting exclusively from the ventrobasalis (thalamic VB nuclei, including the VPL and ventral posterior medial nucleus [VPM]) [22, 23]. Receiving inputs from the PO nucleus, S2L4 neurons process nociceptive stimuli, in response to peripheral nerve injury in mice [24]. When VPL neurons were genetically ablated at birth, S1L4 neurons can also become responsive to nociceptive stimuli, the layer that typically responds to non-nociceptive stimuli [25]. Thus, the identity of S1L4 neurons is not fully committed upon birth, and it is thought that thalamocortical input affects the post-mitotic differentiation of S1L4 neurons [26]. The plasticity of PO neurons halts around postnatal day 10 [25]. S1 plasticity also occurs as a consequence of an injury to the brain [27]. We showed ectopic collateral sprouting from PO neurons in layer 4 of S1 instead of layer 5a, the layer to which PO neuronal axons typically project after hemorrhage to the thalamic VPL nucleus in mice [5] (Fig. 1B). The mice showed a lowered threshold in response to a mechanical stimulus, exhibiting signs of central post-stroke pain (CPSP). CPSP is commonly observed in patients who suffered a stroke, the onset of which is late [28]. The post-stroke brain network reorganization observed over time in stroke patients is considered maladaptive, which relates to pain manifestation [29].

Neuroplastic changes in S1 occur not only after injuries to the thalamus but also after PNS injuries. In the spared nerve injury (SNI) mouse model, which induces chronic pain, activities in S1 layer 5 (L5) pyramidal neurons persistently rise [30] (Fig. 1B). The activity elevation is explained by the effect of less input from the dorsal horn, resulting in the hypoactivity of somatostatin (SOM) neurons, acts in favor of pyramidal neuron hyperactivity. In vivo two-photon imaging experiments have also revealed that L5 pyramidal neurons of the S1 after peripheral nerve injury result in persistent dendritic spine remodeling [31, 32] (Fig. 1B). Meanwhile, the activity of the layer 2/3 neuronal population of S1 acutely increased when mice were injected with CFA into the sole of the foot, inducing an inflammatory type pain. The increase in synchronized neuronal activity in layer 2/3 reduced with N-type calcium ion channel blockers, and the pain level pacified, suggesting that the S1 layer 2/3 neurons mediate the pain level [33]. Whether it is layer 2/3 or layer 5 pyramidal cells, S1 appears to undergo plastic changes relevant to pain pathology associated with peripheral inflammation or peripheral neuropathy. It appears that the projection from the thalamic PO nucleus to the S1 cortex (PO-S1 both glutamatergic neurons, PO^Glu^ -S1^Glu^) is implicated in neuropathic pain induced by either CFA or SNI injury models. Another pathway of the parafascicular thalamic nucleus (PF^Glu^) to the anterior cingulate cortex is implicated in inducing allodynia with depression-like symptoms [34]. Further research is warranted to investigate whether the unique manifestations of pain pathogenesis could be explained by the basis of neuronal circuits and that demonstration of post-injury ectopic somatosensory circuits may further elucidate the differential mechanisms of nociplastic pain and neuropathic pain.

## Dynamic function of microglia in neuroplasticity related to chronic pain

**Fig. 2:**
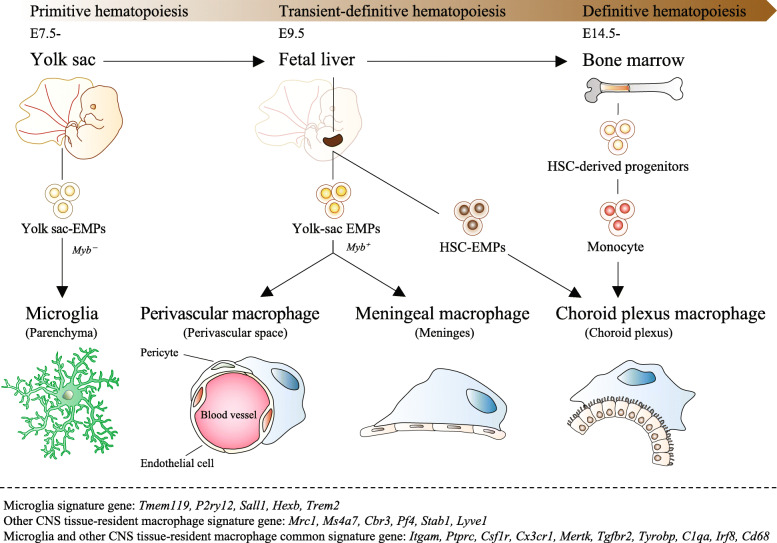
The origin of tissue-resident macrophages (microglia, perivascular/ meningeal/ choroid plexus macrophage) in the CNS. Tissue-resident macrophages of the CNS in mammals are derived from three consecutive hematopoietic systems (primitive hematopoiesis, transient-definitive hematopoiesis, definitive hematopoiesis) during embryogenesis. In mouse primitive hematopoiesis, erythro-myeloid progenitors (EMPs) occur in the yolk sac of extraembryonic tissue during the embryonic period (E7-7.5), and primitive macrophages are produced from these EMPs independently of the transcription factor *Myb* without going through monocytes. Primitive macrophages are then carried to each organ by the circulatory system and engrafted before the formation of the blood-brain barrier. Microglia in the parenchyma of the brain and spinal cord differentiate from these primitive macrophages. Around E9.5, yolk sac-derived EMPs migrate from the yolk sac to the fetal liver and initiate transient-definitive hematopoiesis. Then, in transient-definitive hematopoiesis, monocytes are produced from EMPs in a *Myb*-dependent manner, and the primitive macrophages previously engrafted in each organ are replaced. Since the blood-brain barrier is formed in the brain and infiltration of these monocytes rarely occurs, only microglia present in the parenchyma of the brain and spinal cord are the main origins of primitive macrophages derived in the yolk sac. Around E10.5, hematopoietic stem cells (HSCs) migrate to the fetal liver to start definitive hematopoiesis. HSCs migrate from the fetal liver to the bone marrow before birth and supply the entire line of blood cells throughout life. Choroid plexus macrophages derive from HSC-EMP or blood monocytes. Below the dotted line, microglia and CNS tissue-resident macrophages, and common gene expression patterns are shown

Microglia are known as tissue-resident macrophages of the CNS in the parenchyma of the brain and spinal cord. A genetic fate mapping study using Runx1^cre^ mouse lines revealed that microglia are derived from yolk-sac erythro-myeloid progenitors (EMPs) [35] and that the origin of microglia particularly differs from that of perivascular, meningeal, and choroid plexus macrophages [36, 37] (Fig. 2). The microglial origin can also be separated from that of the other tissue-resident macrophages by the timing the cells migrate to each organ from the time point when the blood-brain barrier is formed [38]. In addition, while other CNS tissue-resident macrophages differentiate in a *Myb* transcription factor-dependent manner, microglia differentiate independently of the *Myb* transcription factor [39], and the gene expression patterns between the two cell types are distinguishable [40–45] (see Fig. 2). The main function of microglia is to physically interact with synapses, debris, and extracellular matrix in phagocytosis [46–56], and to release humoral factors including neurotrophic factors [57–59].

While the microglia play an important role in the development and homeostasis of the nervous system, it has been noted that abnormal microglial hyperactivity may contribute to neuropathology [60–62]. Under various pathological conditions, microglia change their morphology to amoeboid shapes with shortened protrusions and enlarged cell body, indicating microglia are functionally active, from their typical form of ramified shape with multiple finely branched protrusions on the small cell. Reports have shown morphological changes of microglia are associated with pain development [60], some of which could be through the release of humoral factors [32, 63, 64].

After PNS injury, microglial activation accompanied by morphological changes can be observed well beyond the spinal dorsal horn, including in the ventral tegmental area (VTA), the brainstem, and the hippocampus [65–67]. For example, ionized calcium-binding adaptor molecule 1 (Iba1) immunoreactivity, which is indicative of microglial activation, showed that microglia in the VTA increased after a PNS injury in the disruption of the mesolimbic reward circuit [65]. The circuit disruption reduced the dopamine release from the limbic dopaminergic neurons that project from the VTA of the midbrain to the nucleus accumbens (NAc) of the ventral striatum, affecting the emotional aspect of pain. It was, therefore, suggested that such remote microglial functions may underlie morphine-resistant pain sensations. The microglia local to the brainstem also function as a remote control mechanism for thalamic map reorganization that underlies the pathophysiology of neuropathic pain. A unilateral increase in Iba-1-immunoreactivity (activated microglia) was evident in the ipsilateral brainstem principal trigeminal nucleus (Pr5) where axonal sprouting occurred projecting to the thalamic ventral posteromedial nucleus (VPM), but not in the whisker region (barreloids) of the thalamic ventral posteromedial nucleus (VPM) connected to the injury site, the infraorbital nerve, in IONC mouse model. Such IONC induced new axonal innervations from Pr5 to individual VPM neurons, as well as heightened Iba-1 immunoreactivity, accompanied by pain behavior. These injury-induced effects were all suppressed by the systematic microglial depletion with the oral administration of PLX3397, an inhibitor of c-kit and colony-stimulating factor 1 receptors (CSF1Rs) [66], suggesting that microglia is involved in the ectopic sprouting and pain behavior. Another study showed a partial injury to the sciatic nerve activated microglia and induced synaptic changes both remotely in the spinal dorsal horn and hippocampus. Pharmacological inhibition and genetic ablation of microglia result in a reversal of region-specific synaptic plasticity and memory impairment induced by PNS injury. The results revealed how a PNS injury can affect memory with simultaneously evolving chronic pain pathophysiology, at least in part, through microglial function [67]. We have also obtained results that pain can be modulated by the regulation of remote microglial function. Morphologically altered microglia were present for up to 7 days not only in the thalamus, the core damaged by a hemorrhagic injury, but also found in the S1 [5]. The activated microglia in S1 were shown to contribute to ectopic axonal reorganization of the PO-S1L4 circuit, and to modulation of pain behavior. With the administration of a microglial inhibitor, ectopic sprouting and pain behavior were reversed to a non-injury level, suggesting that microglial function in S1 plasticity probably induced nociplastic pain.

Microglia can polarize from the form of ramified shape to the active form of amoeboid shape triggered by a damage to the nervous system, but cell proliferation and the release of various humoral factors by microglia can take place with or without distinctive morphological changes. Microglia without obvious morphological changes have been shown in the amygdala and the somatosensory cortex after nerve injury, and these microglia have been reported to regulate neuropathic pain [32, 68]. Hence, the absence of morphological activation in microglia does not always indicate a lack of functional involvement.

Microglia are said to be derived from the yolk sac; however, in the rise of pain occurrence, it has been shown that circulating monocytes infiltrate into the parenchyma of the spinal dorsal horn and differentiate into the functional microglia-like cells [69]. Chronically, infiltration of these circulating monocytes has also been observed in the central nucleus of the amygdala (CeA) of pain model mice after partial sciatic nerve ligation, and it was shown that anxiety-like behavior was also induced [68]. The study showed that bone marrow (BM)-derived microglia in CeA increased the levels of interleukin (IL)-1b and C-C chemokine receptor type 2 (CCR2). The suppression of BM microglial infiltration with the oral administration of a CCR2 antagonist relieved both pain and anxiety-like behavior; however, the direct microinjection of an IL-1b receptor antagonist into CeA resulted in only a slight improvement in pain behavior, but a complete reduction in anxiety-like behavior. The study showed BM-derived microglia specifically mediated anxiety-like behavior, along with modulating pain development after PNS injury [4, 68].

Other more recent studies have shown, however, the origin of microglia after a nerve injury is unlikely to be derived from circulating monocytes. Studies using mild irradiation, parabiosis mice, and CCR^RFP/+-^CX3CR1^GFP/ +^ mice have indicated that BM-derived microglia were not observed in the parenchyma of the CNS even after nerve injuries [70, 71]. Thus, it is possible the microglia that increase in the CNS parenchyma in the neuropathic pain mouse model are the ones that reside locally. Further studies should show how microglia come about to the damaged tissues.

**Fig. 3:**
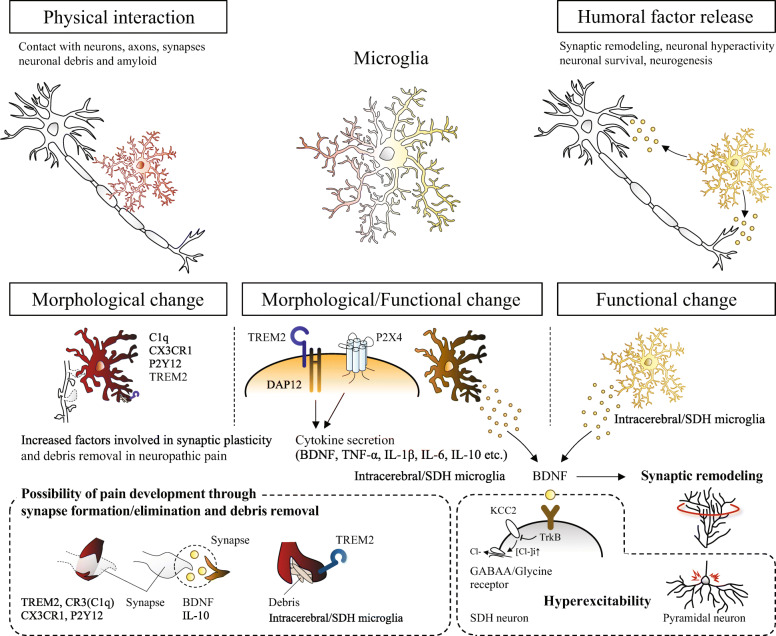
Microglial modulation of neuroplasticity in the spinal dorsal horn and S1 that underlies chronic pain after PNS or CNS injury. TOP: the physical interaction and humoral factor release. BOTTOM: morphological and functional changes in microglia after PNS or CNS injury. Studies showing morphological changes in microglia have not shown specific functions, but have suggested their involvements in the pain development based on the molecular functions. Functional changes in microglia have been suggested to be factors released via TREM2/DAP12 or P2X4 signals. BDNF is one factor shown to be secreted from microglia in the spinal cord and brain after PNS or CNS injury, involved in pain development through synaptic remodeling and inducing hyperexcitability

One of the property changes in microglia is the release of brain-derived neurotrophic factor (BDNF) following PNS injury. Specifically, synaptic remodeling and pyramidal neuron hyperactivity in the S1 cortex induced by nerve injury can be altered by the depletion of microglial-specific BDNF [32]. The specific depletion of BDNF in S1 microglia can also suppress pain behavior developed by PNS injury, suggesting that microglia modulate pain-related neuroplasticity through the release of a growth factor. The mechanism of BDNF released from microglia in pain development is through altering structure and function of synapses that cause increased presynaptic terminals and post-neuron hyperexcitability [4, 64, 72] (Fig. 3). The source of BDNF is likely to be P2X4-expressing microglia [64]. P2X4 is one of the ATP receptors, which could be a molecular mechanism common to spinal cord microglia.

Furthermore, IL-10 secreted from microglia is shown to contribute to synapse formation [73], which increases pain pathology [5]. Hence, IL-10 may be another factor that connects synaptic plasticity and pain development. It has been shown IL-10 is released from TREM2-regulated anti-inflammatory microglia [74, 75]. Cx3CR1, P2Y12, C1q, and TREM2, are also factors that are associated with synapse formation and elimination, or debris removal contributing to pain behavior [47, 50, 51, 53, 76], whose expression levels increase in and around the neuropathic injury areas [5, 77–80]. In the future, further analyses are warranted on how microglia induce and regulate structural and functional synaptic plasticity through these and other factors in pain development.

Together, mounting evidence suggests that cerebral microglia participate in nociplastic changes in distant brain regions to an injury core. Identifying the detailed cellular or molecular microglial functions responsible for inducing neuroplasticity may yield a pharmacological target for treating chronic pain.

## Conclusions

Further elucidation of post-injury ectopic somatosensory circuits may help categorize the mechanisms underlying nociceptive, neuropathic, and nociplastic-type pain. Studies employing cellular and molecular approaches, including neuron type-specific pharmacological molecular inhibition or neuron type-specific genetic ablation, are expected to contribute to the development of a therapy targeting neuroplasticity that induces chronic pain in response to PNS or CNS injury. Among the various functions of microglia, one prominent function seems to be pain regulation in the PNS and CNS. Microglia may directly contribute to synaptic remodeling and altering pain circuits, or contribute indirectly to neuroplasticity through property changes, including the release of growth factors. Hence, further elucidation of the modulatory mechanisms of microglia, including a specific gene-regulating microglial function or cytokines and growth factors released to affect neuroplasticity, may lead to a therapeutic approach that addresses neuroplasticity-relevant chronic pain.

## Data Availability

Not applicable
